# Mechanical circulatory support in patients with cardiogenic shock not secondary to cardiotomy: a network meta-analysis

**DOI:** 10.1007/s10741-021-10092-y

**Published:** 2021-03-06

**Authors:** Stefano Benenati, Matteo Toma, Claudia Canale, Rocco Vergallo, Roberta Della Bona, Davide Ricci, Marco Canepa, Gabriele Crimi, Francesco Santini, Pietro Ameri, Italo Porto

**Affiliations:** 1 Cardiovascular Disease Unit, IRCCS Policlinic Hospital San Martino, Genova, Italy; 2 Department of Internal Medicine, University of Genoa, Genova, Italy; 3 Department of Cardiovascular Sciences, Fondazione Policlinico Universitario Agostino Gemelli IRCCS, Roma, Italy; 4 Cardiac Surgery Unit, IRCCS Policlinic Hospital San Martino, Genova, Italy; 5 Department of Integrated Surgical and Diagnostic Sciences, University of Genova, Genova, Italy

**Keywords:** Cardiogenic shock, Mechanical circulatory support, Extracorporeal membrane oxygenation, Impella, Intra-aortic balloon pump, TandemHeart

## Abstract

To compare the efficacy and safety of different mechanical circulatory support (MCS) devices in CS. A total of 24 studies (7 randomized controlled trials—RCTs—and 17 non-RCTs) involving 11,117 patients were entered in a Bayesian network meta-analysis. The primary endpoint was 30-day mortality. Secondary endpoints were stroke and bleeding (requiring transfusion and/or intracranial and/or fatal). Compared with no MCS, extra-corporeal membrane oxygenation (ECMO) reduced 30-day mortality when used both alone (OR 0.37, 95% CrI 0.15–0.90) and together with the micro-axial pump Impella (OR 0.13, 95% CrI 0.02–0.80) or intra-aortic balloon pump (IABP) (OR 0.19, 95% CrI 0.05–0.63), although the relevant articles were affected by significant publication bias. Consistent results were obtained in a sensitivity analysis including only studies of CS due to myocardial infarction. After halving the weight of studies with a non-RCT design, only the benefit of ECMO + IABP on 30-day mortality was maintained (OR 0.22, 95% CI 0.057–0.76). The risk of bleeding was increased by TandemHeart (OR 13, 95% CrI 3.50–59), Impella (OR 5, 95% CrI 1.60–18), and IABP (OR 2.2, 95% CrI 1.10–4.4). No significant differences were found across MCS strategies regarding stroke. Although limited by important quality issues, the studies performed so far indicate that ECMO, especially if combined with Impella or IABP, reduces short-term mortality in CS. MCS increases the hazard of bleeding.

**Supplementary information:**

The online version contains supplementary material available at 10.1007/s10741-021-10092-y.

## Introduction

Cardiogenic shock (CS) is the most severe manifestation of acute heart failure [1]. The underlying pathophysiology is characterized by left ventricular (LV) dysfunction and elevated filling pressures, systemic hypoperfusion, and neurohormonal activation, triggering and perpetuating one another [2]. As such, treatment of CS is effective only if the cause is promptly removed and/or this vicious spiral is interrupted in the early phases. Indeed, early revascularization has cut mortality in post-myocardial infarction CS (MI-CS) [3]. On the contrary, the increase in cardiac oxygen consumption and afterload induced by inotropic and vasopressor drugs, respectively, outweighs the beneficial effects of these therapies and may eventually lead to higher mortality [4]. Mechanical circulatory support (MCS) improves hemodynamics without the detrimental consequences of pharmacological interventions; therefore, it is generally considered the best non-etiological therapy for CS. Following the introduction of the intra-aortic balloon pump (IABP) in clinical practice, several other devices for MCS, including micro-axial pumps (Impella, Abiomed, Danvers), centrifugal extracorporeal pumps (TandemHeart, LivaNova, London), and extracorporeal membrane oxygenation (ECMO), have become available [5]. Furthermore, emerging evidence suggests that the combination of different MCS is preferable to the use of a single device to support hemodynamics and decompress the LV in the meanwhile [6].

However, studies about the efficacy-safety profile of MCS in CS have produced discordant results, and the choice of the type of MCS remains largely based on individual expertise, costs, and local availability. The discrepancies in the literature about MCS in CS at least in part stem from the substantial differences existing between the two most common presentations of CS, i.e., MI-CS and post-cardiotomy CS, and from the variable duration of the follow-up, ranging from the length of the hospitalization to 2 weeks or 1 month. Clearly, the longer is the time window during which mortality is evaluated, the stronger are the conclusions on the effect of MCS on survival. These shortcomings have been carried over in the meta-analyses published so far on the topic [6–12]. Furthermore, the statistical approach adopted for these meta-analyses did not allow drawing comparisons between different types of MCS when head-to-head primary studies were not available.

To overcome these limitations, we performed a network meta-analysis to obtain a comprehensive view of the data supporting the different MCS strategies in patients with non-post-cardiotomy CS.

## Methods

This meta-analysis was registered on PROSPERO (CRD42020189859) and followed the Preferred Reporting Items for Systematic Reviews and Meta-Analysis (PRISMA) recommendations [13] (Appendix Table S1).

### Study selection, data extraction, quality assessment, and endpoint definition

Between February and April 2020, we conducted a systematic search using Pubmed/MEDLINE, Web of Science, Embase, Cochrane, ClinicalTrials.gov, and TCTMD (https://www.tctmd.com) using the following terms: “cardiogenic shock,” “intra-aortic balloon pump,” “Impella,” “extracorporeal membrane oxygenation,” “ECMO,” and “TandemHeart” (full search strategy in Appendix Table S2). Previously published meta-analyses and reviews on the topic were also examined, and references of the included studies were screened using a “snowball” approach. Abstract and presentations from the major congresses of the main Cardiology Scientific Societies (American College of Cardiology, European Society of Cardiology, American Heart Association, and Transcatheter Cardiovascular Therapeutics) were also evaluated.

We selected the articles comparing MCS vs. no MCS or different MCS strategies in patients with CS. Studies enrolling post-cardiotomy CS, providing insufficient outcome data or published in languages other than English, were excluded. When data for a certain endpoint were insufficient, the treatment arm was excluded from the analysis for that outcome, in order to avoid misleading results. Three independent investigators (SB, MT, and CC) screened the titles and abstracts and subsequently read the full texts of candidate articles to confirm whether they fulfilled the criteria for inclusion. Next, information was extracted on (a) study characteristics (inclusion and exclusion criteria, treatment strategy in the intervention and control arms, number of patients per arm, year of publication), (b) patients’ baseline characteristics, and (c) outcome measures. The Cochrane Collaboration’s Tool [14] and the ROBINS-I Tool [15] were applied to assess the risk of bias.

The primary endpoint was 30-day mortality. Secondary endpoints were (1) bleeding, defined as bleeding requiring transfusion and/or intracranial hemorrhage and/or fatal bleeding according to the data provided by the studies, and (2) stroke, as defined in each study.

### Statistical analysis

The effects of different MCS devices on the primary and secondary endpoints were compared by means of a random-effect Bayesian meta-analysis, integrating direct and indirect evidence and assuming no discrepancy (i.e., evidence consistency). Random-effect models were used to take into account the heterogeneity between studies. Odds ratios (OR) and 95% credible intervals (CrI) were obtained using a Markov-chain Monte Carlo simulation, by running four chains of 30,000 interactions after a burn-in of 10,000. Heterogeneity was measured through the *I*^2^ statistic, with < 25%, 25–50%, and > 50% *I*^2^ indicating, respectively, low, moderate, or high heterogeneity [16]. Treatments were also ranked using the surface under the cumulative ranking curve (SUCRA), which allows quantifying the probability of each treatment to be the best by attributing a probability from zero to one, with highest SUCRA values suggesting better-performing treatments.

Publication bias was assessed using comparison-adjusted funnel plots of effect size against standard error and Egger’s tests. Small studies tend to give more emphasis to the effectiveness of a treatment, to the point that it may result the best in network meta-analysis because it has been mostly investigated in small cohorts. To verify this hypothesis, treatments were ordered from the most effective to the least effective [17]. Evidence consistency was evaluated through the node-splitting technique [18].

Sensitivity analyses were performed on the primary and secondary endpoints using the frequentist approach and including only studies of MI-CS. Furthermore, we re-analyzed the selected studies after halving the weight of those with non-randomized controlled trial (RCT) design, in order to account for the lower quality of non-RCT data. Finally, we performed a network meta-regression on the primary endpoint by grouping the selected articles by 5-year periods of publication (1997–2001, 2002–2006, 2007–2011, 2012–2016, 2017–2020), in order to determine the influence, if any, of different times in which the studies were conducted. The goodness of fit of the regression model was then compared with that of the original model by means of the Deviance Information Criterion (DIC), with differences above 3 points considered meaningful [18].

The analysis was carried out using R (The R Foundation for Statistical Computing, version 3.6.2).

## Results

Our search strategy yielded 4590 articles, of which 24 met the selection criteria and were thus analyzed (Appendix Figure S1 shows the PRISMA flowchart). Seven were RCTs and 17 observational studies; the list and corresponding references are given in Appendix Table S3. Among the included articles, 10 compared IABP vs. no MCS, 6 IABP vs. Impella, 3 IABP vs. TandemHeart, 2 ECMO vs. IABP, and 2 ECMO vs. ECMO + IABP, whereas each remaining direct comparison was based on a single study. The overall population amounted to 11,117 subjects, with 845 (7.6%), contributing with a total of 337 events, being from RCTs.

**Fig. 1:**
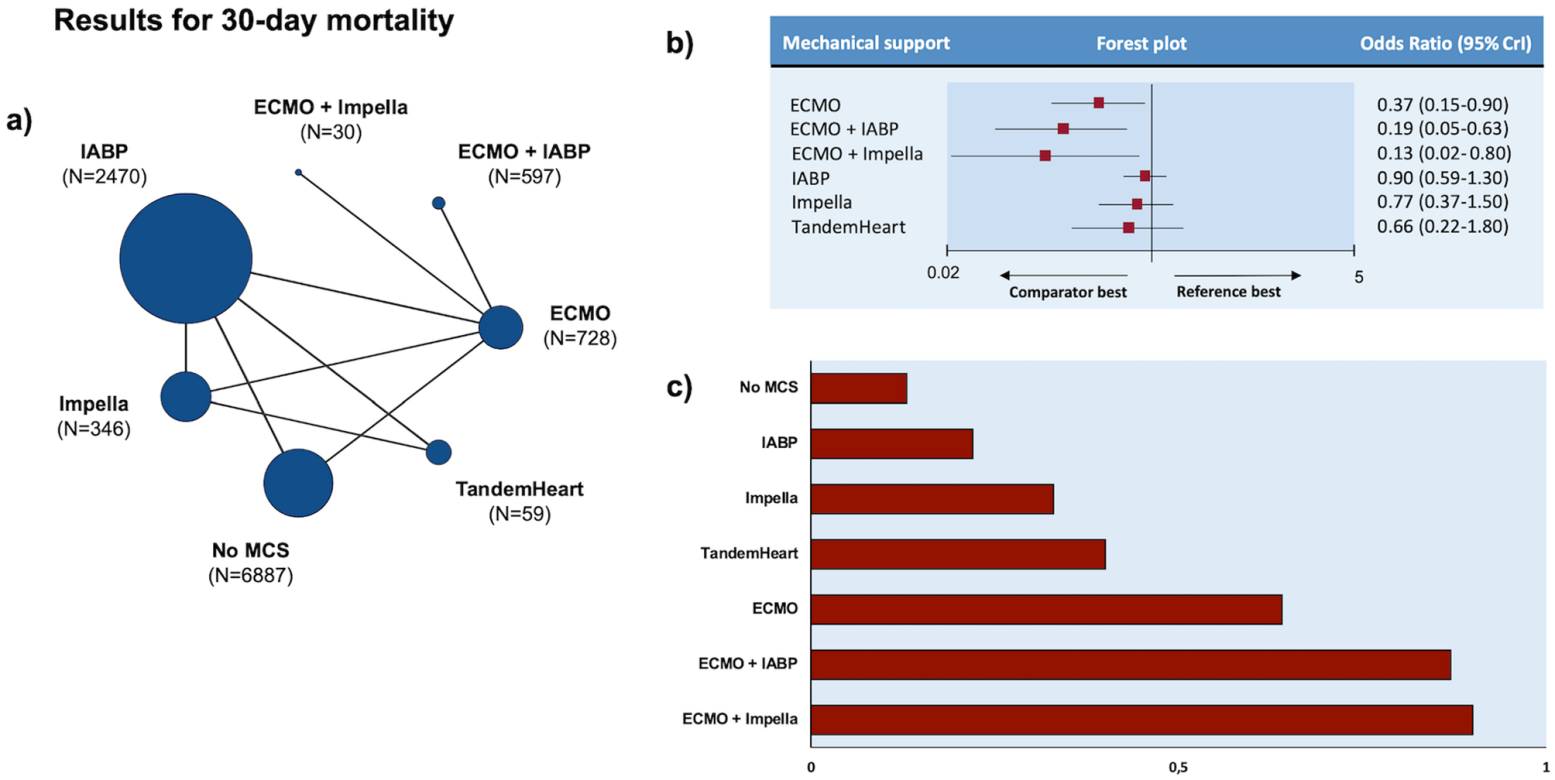
Analysis of 30-day mortality. **a** Treatments network. Each treatment is represented by a colored circle (node) of size proportional to the number of studies in which it was evaluated. The number of patients receiving each treatment is reported in parentheses. **b** Forest plot. Reference is “no MCS”, whereas comparators are shown on the left part of the panel. **c** Treatment ranking according to SUCRA values. Red bars indicate SUCRA values on a scale from 0 to 1. Longer bars (i.e., higher SUCRA values) indicate better-performing treatment. Abbreviations: CrI credible interval, ECMO extra-corporeal membrane oxygenation, IABP intra-aortic balloon pump, MCS mechanical circulatory support

The number of patients treated with each MCS strategy is displayed in Fig. 1a. The main features of each study and the patients’ baseline characteristics are reported in Appendix Tables S4-S5. Median age, as calculated by the values provided in 21 (84%) of the selected articles, was 65 years. With the exception of the control arm of one investigation (20), male gender was always more frequent than female one. Information about body mass index, cardiovascular risk factors, and renal function was largely missing (Appendix Table S5). Remarkably, 20 studies (*N* = 9,864, 88.7% of total) included only MI-CS patients. Bias assessment is reported in Appendix Table S6. Overall, the risk of bias was low for RCTs, whereas it ranged from moderate to high for non-RCT studies.

### Primary endpoint

Treatment network for the primary endpoint is shown in the Fig. 1a: 6887 (62% of total) patients received no MCS. Among those, 3165 (46%) died at 30 days. IABP was the most frequent MCS, accounting for 2470 (22%) subjects. Compared with no MCS, ECMO reduced 30-day mortality when used both alone (OR 0.37, 95% CrI 0.15–0.90) and in combination with another MCS (ECMO + Impella vs. no MCS: OR 0.13, 95% CrI 0.02–0.80; ECMO + IABP vs no MCS: OR 0.19, 95% CrI 0.05–0.63). There were no significant differences in 30-day mortality between no MCS and other MCS strategies (Fig. 1b). When treatment was ranked according to SUCRA values, the combination of ECMO + Impella was the best, followed by ECMO + IABP and ECMO alone (Fig. 1c).

### Secondary endpoints

**Fig. 2:**
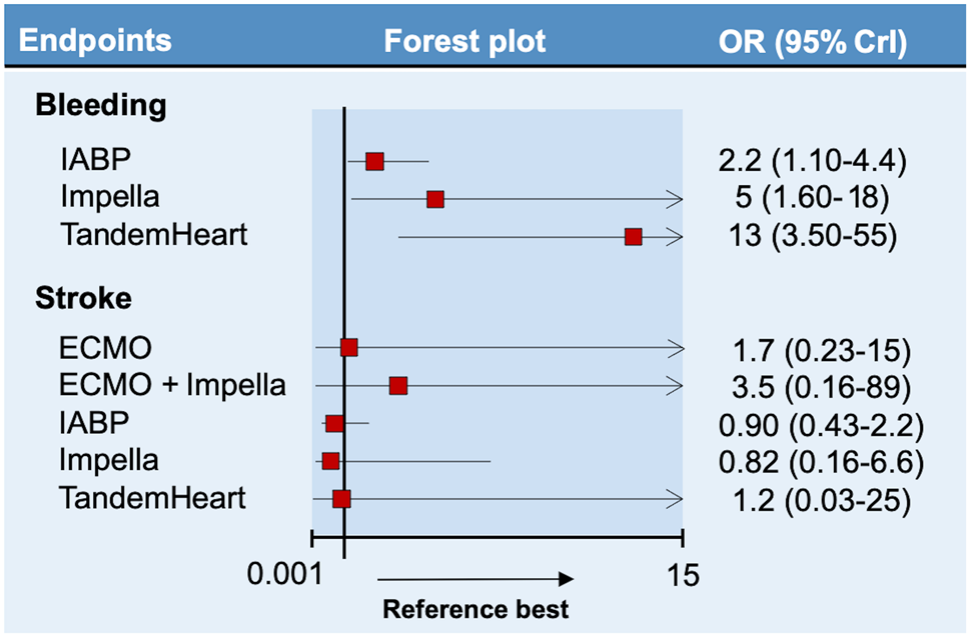
Forest plots for bleeding and stroke. Reference is “no MCS,” whereas comparators are shown on the left part of the figure. When the upper limit of the credible interval (CrI) exceeds the scale of the plot, an arrow is displayed. Abbreviations: OR odds ratio, ECMO extra-corporeal membrane oxygenation, IABP intra-aortic balloon pump

Treatment networks for the secondary endpoints are shown in Appendix Figure S2. Twelve studies reported the incidence of bleeding for a total 8856 patients. Due to the lack of outcome data, the network for bleeding did not include ECMO, ECMO + Impella, and ECMO + IABP. Compared with no MCS, TandemHeart yielded the highest risk of bleeding (OR 13, 95% CrI 3.50–59), followed by Impella (OR 5, 95% CrI 1.60–18) and IABP (OR 2.2, 95% CrI 1.10–4.4) (Fig. 2).

The network for stroke included 14 studies and 9330 patients distributed between all treatments strategies except for ECMO + IABP. Results were neutral across all MCS types, as shown in Fig. 2.

### Publication bias, heterogeneity, and node-split analysis

Comparison-adjusted funnel plots were suggestive of publication bias for the primary endpoint and for the stroke secondary endpoint, indicating that a small-study effect might have favored the most effective treatments (Appendix Figure S3). This was confirmed by the Egger’s test (*p* < 0.01 for the primary endpoint and *p* = 0.02 for the stroke secondary endpoint). Conversely, no hint of publication bias was noted for bleeding (Appendix Figure S3). Heterogeneity was generally high, as suggested by global and per-comparisons I^2^ (Appendix Table S7). Node-split analyses are presented in Appendix Figure S4. There was a discrepancy between direct and indirect comparisons between TandemHeart and Impella for the primary endpoint, although the *p* value for inconsistency was non-significant. No clue of inconsistency was noted with respect to other comparisons. As for bleeding, node-split analysis was available only for the comparison between TandemHeart and Impella because (1) the network is not a closed loop and (2) the comparison between IABP, Impella, and TandemHeart relied on a three-arm study, thus splitting other nodes would not result in an adequate network (i.e., containing studies that did not include both the split nodes) [18]. The analysis showed no hint of inconsistency for this endpoint. No inconsistency was noted with respect to stroke.

### Sensitivity analyses

Results of the sensitivity analysis restricted to MI-CS were in agreement with the main analysis, although the lowest odds of 30-day mortality was associated with ECMO + IABP (OR 0.10, 95% CrI 0.017–0.57) (Online Figure S5). Due to the lack of outcome data, ECMO, ECMO + Impella, ECMO + IABP, and TandemHeart were not included in the bleeding network, nor were ECMO + IABP and TandemHeart in the stroke network. The effects of the different MCS strategies on the risk of stroke in MI-CS were comparable to those found in CS in general (Appendix Figure S5). As for bleeding, point estimates were in the same direction as in the main analysis; however, the risk of bleeding with both IABP and Impella was no longer significant (Appendix Figure S5). The frequentist analysis yielded similar results for all three endpoints as compared with the Bayesian one (Appendix Figure S6).

After weight-adjustment according to study design, 30-day mortality was less likely with ECMO and ECMO + Impella than with no MCS, but not to a significant extent (ECMO vs no MCS: OR 0.40, 95% CI 0.15–1; ECMO + Impella vs. no MCS: OR 0.14, 95% CI 0.017–1) (Appendix Figure S7). Similarly, the increase of bleeding with IABP was non-significant. The other results for this sensitivity analysis were in line with the main analysis (Appendix Figure S7).

Meta-regression revealed no significant influence of the 5-year periods of publication on effect estimate (beta coefficient: 0.60, 95% CrI − 0.10–1.30). The regression model fit did not meaningfully differ from the original model (DIC of the original model = 91.7; DIC of the regression model = 91).

## Discussion

The role played by MCS in the treatment of CS is still debated. From the pathophysiological standpoint, MCS is preferable to drugs with inotropic and vasopressor activity, since it compensates for the reduced output of the failing heart and maintains systemic perfusion [19]. However, the overall impact of MCS on hard clinical endpoints in RCTs has been disappointing [20–22]. In fact, the use of IABP has been downgraded to a class IIa level of evidence (LoE) B and IIa LoE C recommendations, respectively, in the US and European guidelines [23, 24] following the results of the IABP-SHOCK II RCT [20, 25]. Nevertheless, it has been pointed out that at least some RCTs were not adequately powered to detect differences in mortality. It has also been argued that the population enrolled in RCTs was not representative of the patient phenotype that would actually benefit from MCS. RCTs might have missed the so called “sweet spot,” i.e. ,the best timing to implant a device for MCS before it is no longer effective in modifying the course of CS and, thereby, becomes futile [26, 27]. Therefore, a better estimate of the impact of MCS in CS may be obtained by pooling together the data from RCTs and observational studies. Here, we did so by performing a network meta-analysis. This methodology, which has only recently introduced in the field [28], allowed us to compare all the MCS strategies for CS on a solid statistical basis. By contrast, the classical meta-analytic approach does not measure the relative efficacy and safety of interventions that were not directly compared in prior studies.

Our work is also original for other aspects. We restricted the population of interest to the one affected by CS not secondary to cardiotomy, in order to avoid a selection bias in favor of ECMO, which is the most adopted MCS in the surgical setting. Moreover, we rigorously assessed mortality at 30 days, which in our opinion is a stronger efficacy outcome compared with in-hospital or 2-week mortality and gives a clear-cut measure of the effect that MCS may have in clinical practice.

Our meta-analysis suggests that ECMO reduces 30-day mortality of patients with CS, especially in combination with Impella or IABP. ECMO is a powerful MCS, which replaces the function of both ventricles and provides a flow of up to 4–6 l/min [29]. However, it injects oxygenated blood against the LV, increasing the afterload, worsening LV filling pressures, and favoring pulmonary congestion [29, 30]. This drawback can be overcome by surgical venting or by combining ECMO with another percutaneous MCS. Thus, unloading of the LV is a mechanistic explanation for the further decrease in mortality we found with the association of ECMO with Impella or IABP, as compared with ECMO alone. Indeed, Impella was shown to lower pulmonary capillary wedge pressure when added to ECMO (32), with hemodynamic and clinical results comparable to those of surgical venting [31]. Moreover, in a recent meta-analysis focused on the effects of combining IABP with ECMO, this approach significantly reduced short-term mortality in MI-CS [6]. A double-MCS strategy also offers the possibility of de-escalating the support to the LV, allowing a smoother transition toward weaning or destination therapy [32]. Unfortunately, we could not retrieve sufficient information about surgical venting in patients who received ECMO alone. Therefore, the superiority of ECMO + Impella or IABP might be due to inadequate venting in the arms treated with ECMO alone, rather than to the advantage of adding a second MCS device.

The present systematic review also revealed a significant publication bias, which must be taken into consideration when discussing the apparent efficacy of ECMO (with or without Impella or IABP), but not of the other types of MCS on mortality in CS. No RCT has evaluated ECMO and ECMO + Impella so far, and the benefit of these strategies for MCS was no longer significant after adjusting for study design.

It should be also noted that most of the studies included in the analysis were with Impella 2.5, when newer and more powerful Impella devices are available. Interestingly, observational investigations showed a similar survival with ECMO and Impella CP after adjusting for patients’ risk profile [33]. Hence, our results cannot be generalized to the whole Impella family. The ongoing Danish Cardiogenic Shock trial will shed light regarding the efficacy of Impella CP in MI-CS [34].

In this meta-analysis, there was a gradient in bleeding risk, with TandemHeart being the worst strategy and both Impella and IABP significantly increasing the odds of bleeding compared with no MCS. TandemHeart and Impella require large-bore arterial cannulation, which can be complicated by access-site bleeding. Furthermore, both TandemHeart and Impella require anticoagulation with unfractionated heparin, which increases the likelihood of bleeding. Although not mandatory, anticoagulation is also often adopted for IABP and this may be the reason why this device was also associated with a higher risk of bleeding. Due to the lack of data, we could not assess bleeding with ECMO. However, the considerations above hold true for this device, which has been associated with a significant and prognostically meaningful risk of bleeding [35]. Indeed, in 20 studies including 1866 subjects, the rate of ECMO-related bleeding was 41% [36].

CS is a complex syndrome, and our findings may not be valid for specific scenarios. For instance, we could not assess the efficacy of the different devices for MCS according the severity and duration of CS, or to the absence or presence of right ventricular failure. Patients treated with Impella and presenting with an INTERMACS class II have better survival than those in INTERMACS class I [37]. Moreover, Impella is a pure LV MCS and, as such, is not suitable in CS with biventricular failure [38]. Comorbidities and concomitant medications are other factors that influence the performance of MCS in CS, but we could not take into account.

Local expertise is another factor that is key for the successful management of CS, but we could not gauge. IABP is still widely used for incipient CS in centers where other types of MCS are not available or rarely employed [38]. Conversely, Impella and ECMO need dedicated personnel, who handle the devices in the optimal manner only after a learning curve, and a structured network of spoke and hub centers [2].

Favoring ECMO and, to a greater extent, its combinations with an unloading-dedicated device, our results prove that times would be ripe to test these strategies in adequately powered RCTs. Focusing on specific etiologies (e.g. non post-cardiotomy CS, as we did in the present work) and phenotypes (e.g., biventricular vs. LV isolated dysfunction) and standardizing the endpoints definitions would be necessary to make future studies more informative.

### Limitations

This study-level meta-analysis was based on articles largely lacking information about patients’ characteristics, whose influence on the estimates was therefore hardly assessable. However, a sensitivity analysis was run for MI-CS, and a network meta-regression was used to evaluate the impact of the period of publication.

Included studies were largely different with respect to design, outcomes definitions, enrolled populations, and treatment strategies. To account for this heterogeneity, the analysis was conducted using random-effect models. Many studies were observational, often including a small sample size. In particular, only 30 patients in one study [18] were treated with ECMO + Impella. Although this implicates a poor quality of evidence, we performed a sensitivity analysis according to study design and time of publication and accurately examined the influence of publication bias.

Other important clinical endpoints influence the clinical course of patients undergoing MCS, such as limb ischemia. Other authors included this complication in their meta-analysis, based on very limited data largely derived from a single study [10]. Instead, we preferred not carrying out such analysis, as it would rely on insufficient information. Similarly, data were too limited to take into consideration other possible complications of MCS, which however may have a devastating clinical impact, i.e., limb amputation, compartment syndrome and fasciotomy, non-stroke neurological issues, and infection.

Finally, data on the duration of CS before MCS implantation were absent in most articles.

## Conclusions

Data from RCTs and observational studies indicate that, in CS with various etiologies, ECMO significantly decreases short-term mortality as compared with other types of MCS or no support, especially when used in association with Impella or IABP. This finding should be considered hypothesis-generating and inform larger and adequately powered RCTs. The risk of bleeding is enhanced by MCS, but data on this outcome with ECMO are very limited.

## Supplementary Information

Below is the link to the electronic supplementary material.

Supplementary file1 (DOCX 6778 KB)

## Data Availability

Under appropriate request to the corresponding author.

## Code Availability

Under appropriate request to the corresponding author.
