# Supplementary material for: Mechanical circulatory support in patients with cardiogenic shock not secondary to cardiotomy: a network meta-analysis
Source: Heart Fail Rev. 2021 Mar 6;27(3):927–34. doi: 10.1007/s10741-021-10092-y (PMC9033692; doi:10.1007/s10741-021-10092-y)

**Mechanical circulatory support in patients with cardiogenic shock not secondary to cardiotomy: a network meta-analysis**

Stefano Benenati, Matteo Toma, Claudia Canale, Rocco Vergallo, Roberta Della Bona, Davide Ricci, Marco Canepa, Gabriele Crimi, Francesco Santini, Pietro Ameri, Italo Porto

**Supplementary Appendix**

**Table of contents**

**Appendix Table S1. PRISMA checklist**

**Appendix Table S2. Full electronic search in MEDLINE/Pubmed database through 30^th^ April, 2020**

**Appendix Figure S1. PRISMA flowchart**

**Appendix Table S3. List of the studies included meta-analysis**

**Appendix Table S4. Characteristics of the included studies**

**Appendix Table S5. Patients’ baseline characteristics**

**Appendix Table S6. Risk of bias assessment. Risk of bias of a) individual randomized clinical trials by Cochrane risk assessment tool and b1-2) individual observational studies by ROBINS-I tool**

**Appendix Figure S2. Treatment network for the secondary endpoints**

**Appendix Figure S3. Comparison-adjusted funnel plots and Egger’s tests**

**Appendix Table S7. Per-comparisons and global heterogeneity according to I^2^**

**Appendix Figure S4. Node-spit analyses**

**Appendix Figure S5. Sensitivity analysis including only studies of MI-CS**

**Appendix Figure S6. Sensitivity analyses using the frequentist approach**

**Appendix Figure S7. Sensitivity analyses after underweighting of non-RCT studies**

**Appendix Table S1. PRISMA checklist**

| **Section/topic** | **#** | **Checklist item** | **Reported on page #** |
| --- | --- | --- | --- |
| **TITLE** | | |  |
| Title | 1 | Identify the report as a systematic review incorporating a network meta-analysis (or related form of meta-analysis). | 1 |
| **ABSTRACT** | | |  |
| Structured summary | 2 | Provide a structured summary including, as applicable: 1) Background: main objectives; 2)Methods: data sources; study eligibility criteria, participants, and interventions; study appraisal; and synthesis methods, such as network meta-analysis; 3) Results: number of studies and participants identified; summary estimates with corresponding  confidence/credible intervals; treatment rankings may also be discussed. Authors may choose to summarize pairwise comparisons against a chosen treatment included in their analyses for brevity. 4) Discussion/Conclusions: limitations; conclusions and implications of findings. 5) Other: primary source of funding; systematic review registration number with registry name. | 3 |
| **INTRODUCTION** | | |  |
| Rationale | 3 | Describe the rationale for the review in the context of what is already known, including mention of why a network meta-analysis has been conducted. | 4 |
| Objectives | 4 | Provide an explicit statement of questions being addressed with reference to participants, interventions, comparisons, outcomes, and study design (PICOS). | 4 |
| **METHODS** | | |  |
| Protocol and registration | 5 | Indicate if a review protocol exists, if and where it can be accessed (e.g., Web address), and, if available, provide registration information including registration number. | CRD42020189859 |
| Eligibility criteria | 6 | Specify study characteristics (e.g., PICOS, length of follow-up) and report characteristics (e.g., years considered, language, publication status) used as criteria for eligibility, giving rationale. Clearly describe eligible treatments included in the treatment network, and note whether any have been clustered or merged into the same node (with justification). | 4-5 |
| Information sources | 7 | Describe all information sources (e.g., databases with dates of coverage, contact with study authors to identify additional studies) in the search and date last searched. | 4-5 |
| Search | 8 | Present full electronic search strategy for at least one database, including any limits used, such that it could be repeated. | Appendix Table S2 |
| Study selection | 9 | State the process for selecting studies (i.e., screening, eligibility, included in systematic review, and, if applicable, included in the meta-analysis). | 4-5 |
| Data collection process | 10 | Describe method of data extraction from reports (e.g., piloted forms, independently, in duplicate) and any processes for obtaining and confirming data from investigators. | 4-5 |
| Data items | 11 | List and define all variables for which data were sought (e.g., PICOS, funding sources) and any assumptions and simplifications made. | 4-5 |
| Geometry of the network | S1 | Describe methods used to explore the geometry of the treatment network under study and potential biases related to it. This should include how the evidence base has been graphically summarized for presentation, and what characteristics were compiled and used to describe the evidence base to readers. | 4-6 |
| Risk of bias within individual studies | 12 | Describe methods used for assessing risk of bias of individual studies (including specification of whether this was done at the study or outcome level), and how this information is to be used in any data synthesis. | Appendix Table S6 |
| Summary measures | 13 | State the principal summary measures (e.g., risk ratio, difference in means). Also describe the use of additional summary measures assessed, such as treatment rankings and surface under the cumulative ranking curve (SUCRA) values, as well as modified approaches used to present summary findings from meta-analyses. | 5 |
| Planned method of analysis | 14 | Describe the methods of handling data and combining results of studies for each network meta-analysis. This should include, but not be limited to: handling of multi-group trials; Selection of variance structure; Selection of prior distributions in Bayesian analyses; and Assessment of model fit. | 5 |
| Assessment of inconsistency | S2 | Describe the statistical methods used to evaluate the agreement of direct and indirect evidence in the treatment network(s) studied. Describe efforts taken to address its presence when found. | 5 |
| Risk of bias across studies | 15 | Specify any assessment of risk of bias that may affect the cumulative evidence (e.g., publication bias, selective reporting within studies). | Appendix Table S6 |
| Additional analyses | 16 | Describe methods of additional analyses if done, indicating which were pre-specified. This may include, but not be limited to, the following: sensitivity or subgroup analyses; Meta-regression analyses; Alternative formulations of the treatment network; and Use of alternative prior distributions for Bayesian analyses (if applicable). | 5 |
| **RESULTS** | | |  |
| Study selection | 17 | Give numbers of studies screened, assessed for eligibility, and included in the review, with reasons for exclusions at each stage, ideally with a flow diagram. | 6 |
| Presentation of network structure | S3 | Provide a network graph of the included studies to enable visualization of the geometry of the treatment network. | 6-7 |
| Summary of network geometry | S4 | Provide a brief overview of characteristics of the treatment network. This may include commentary on the abundance of trials and randomized patients for the different interventions and pairwise comparisons in the network, gaps of evidence in the treatment network, and potential biases reflected by the network structure. | 6-7 |
| Study characteristics | 18 | For each study, present characteristics for which data were extracted (e.g., study size, PICOS, follow-up period) and provide the citations. | Appendix Table S3 |
| Risk of bias within studies | 19 | Present data on risk of bias of each study and, if available, any outcome level assessment. | 6-9 |
| Results of individual studies | 20 | For all outcomes considered (benefits or harms), present, for each study: 1) simple summary data for each intervention group, and 2) effect estimates and confidence intervals. Modified approaches may be needed to deal with information from larger networks. | 6-9 |
| Synthesis of results | 21 | Present results of each meta-analysis done, including confidence/credible intervals. In larger networks, authors may focus on comparisons versus a particular comparator (e.g., placebo or standard care), with full findings presented in an appendix. League tables and forest plots may be considered to summarize pairwise comparisons. If additional summary measures were explored (such as treatment rankings), these should also be presented. | 6-9 |
| Exploration of inconsistency | S5 | Describe results from investigations of inconsistency. This may include such information as measures of model fit to compare consistency and inconsistency models, P values from statistical tests, or summary of inconsistency estimates from different parts of the treatment network. | 6-9 |
| Risk of bias across studies | 22 | Present results of any assessment of risk of bias across studies for the evidence base being studied. | 6-9 |
| Results of additional analyses | 23 | Give results of additional analyses, if done (e.g., sensitivity or subgroup analyses, meta-regression analyses, alternative network geometries studied, alternative choice of prior distributions for Bayesian analyses, and so forth). | 6-9 |
| **DISCUSSION** | | |  |
| Summary of evidence | 24 | Summarize the main findings, including the strength of evidence for each main outcome; consider their relevance to key groups (e.g., health care providers, researchers, and policymakers). | 7-11 |
| Limitations | 25 | Discuss limitations at study and outcome level (e.g., risk of bias), and at review level (e.g., incomplete retrieval of identified research, reporting bias). Comment on the validity of the assumptions, such as transitivity and consistency. Comment on any concerns regarding network geometry (e.g., avoidance of certain comparisons). | 11-12 |
| Conclusions | 26 | Provide a general interpretation of the results in the context of other evidence, and implications for future research. | 12 |
| **FUNDING** | | |  |
| Funding | 27 | Describe sources of funding for the systematic review and other support (e.g., supply of data); role of funders for the systematic review. This should also include information regarding whether funding has been received from manufacturers of treatments in the network and/or whether some of the authors are content experts with professional conflicts of interest that could affect use of treatments in the network. | No funding received |

**Appendix Table S2. Full electronic search in the MEDLINE/Pubmed database through April, 30^th^ 2020**

| **Research** | **Query** | **Items found** |
| --- | --- | --- |
| 1 | Cardiogenic shock | 14733 |
| 2 | Mechanical circulatory support | 9590 |
| 3 | Intra-aortic balloon pump | 5678 |
| 4 | Extracorporeal membrane oxygenation | 14162 |
| 5 | Impella | 772 |
| 6 | TandemHeart | 185 |
| 7 | Cardiogenic shock AND mechanical circulatory support | 1043 |
| 8 | Cardiogenic shock AND Impella | 436 |
| 9 | Cardiogenic shock AND intra-aortic ballon pump | 1639 |
| 10 | Cardiogenic shock AND extracorporeal membrane oxygenation | 1467 |
| 11 | Cardiogenic shock AND TandemHeart | 119 |

**Appendix Figure S1. PRISMA flowchart**


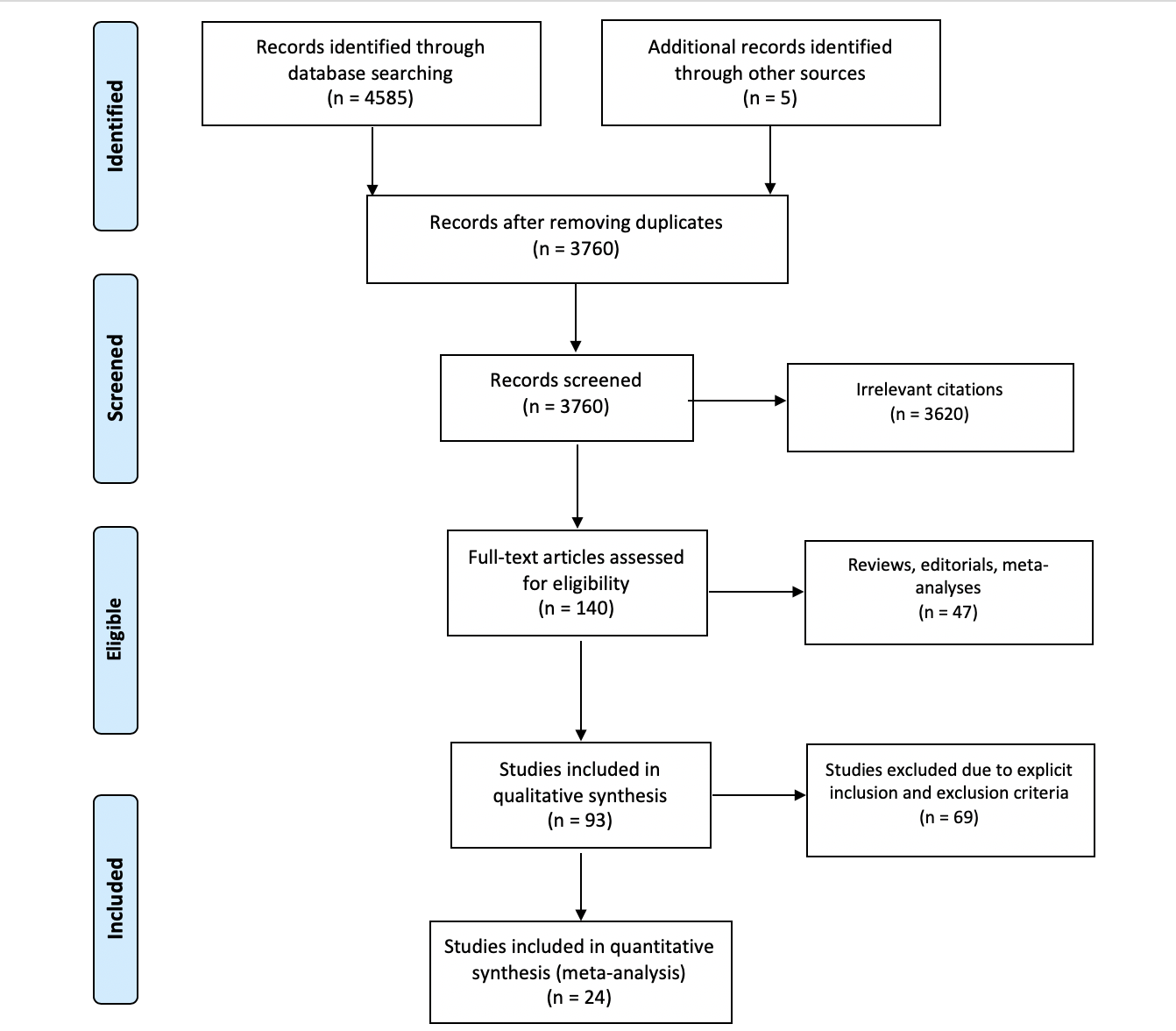


**Appendix Table S3. List of the studies included in the meta-analysis**

| ALUSHI (1) | ANDERSON (2) | ASO (3) | BRUNNER (4) | BURKHOFF (5) |
| --- | --- | --- | --- | --- |
| DZIWIERZ (6) | GU (7) | HAWRANEK(8) | IABP SHOCK II (9) | IMPRESS (10) |
| IQBAL (11) | ISAR SHOCK (12) | KARAMI (13) | KOVACK(14) | MANZO-SILBERMAN (15) |
| OHMAN (16) | OVERTCHOUK (17) | PATEL(18) | SATTLER(19) | SCWARTZ(20) |
| SHARAGE(21) | SHEU (22) | SJAUW (23) | THIELE (24) |  |

**References**

1. Alushi B, Douedari A, Froehlig G, Knie W, Wurster TH, Leistner DM, et al. Impella versus IABP in acute myocardial infarction complicated by cardiogenic shock. Open Hear. 2019;

2. Anderson RD, Ohman EM, Holmes DR, Col J, Stebbins AL, Bates ER, et al. Use of intraaortic balloon counterpulsation in patients presenting with cardiogenic shock: Observations from the GUSTO-I study. Journal of the American College of Cardiology. 1997.

3. Aso S, Matsui H, Fushimi K, Yasunaga H. The Effect of Intraaortic Balloon Pumping under Venoarterial Extracorporeal Membrane Oxygenation on Mortality of Cardiogenic Patients: An Analysis Using a Nationwide Inpatient Database. Crit Care Med. 2016;

4. Brunner S, Guenther SPW, Lackermair K, Peterss S, Orban M, Boulesteix AL, et al. Extracorporeal Life Support in Cardiogenic Shock Complicating Acute Myocardial Infarction. Journal of the American College of Cardiology. 2019.

5. Burkhoff D, Cohen H, Brunckhorst C, O’Neill WW. A randomized multicenter clinical study to evaluate the safety and efficacy of the TandemHeart percutaneous ventricular assist device versus conventional therapy with intraaortic balloon pumping for treatment of cardiogenic shock. Am Heart J. 2006;

6. Dziewierz A, Siudak Z, Rakowski T, Kleczyński P, Zasada W, Dudek D. Impact of intra-aortic balloon pump on long-term mortality of unselected patients with ST-segment elevation myocardial infarction complicated by cardiogenic shock. Postep w Kardiol Interwencyjnej. 2014;

7. Gu J, Hu W, Xiao H, Feng X, Chen Y, Zhang D. Intra-aortic balloon pump improves clinical prognosis and attenuates C-reactive protein level in acute STEMI complicated by cardiogenic shock. Cardiology. 2010;

8. Hawranek M, Gierlotka M, Pres D, Zembala M, Gąsior M. Nonroutine Use of Intra-Aortic Balloon Pump in Cardiogenic Shock Complicating Myocardial Infarction With Successful and Unsuccessful Primary Percutaneous Coronary Intervention. JACC Cardiovasc Interv. 2018;

9. Thiele H, Zeymer U, Neumann FJ, Ferenc M, Olbrich HG, Hausleiter J, et al. Intraaortic balloon support for myocardial infarction with cardiogenic shock. N Engl J Med. 2012;

10. Ouweneel DM, Eriksen E, Sjauw KD, van Dongen IM, Hirsch A, Packer EJS, et al. Percutaneous Mechanical Circulatory Support Versus Intra-Aortic Balloon Pump in Cardiogenic Shock After Acute Myocardial Infarction. J Am Coll Cardiol. 2017;

11. Iqbal MB, Robinson SD, Ding L, Fung A, Aymong E, Chan AW, et al. Intra-aortic balloon pump counterpulsation during primary percutaneous coronary intervention for ST-elevation myocardial infarction and Cardiogenic shock: Insights from the British Columbia Cardiac Registry. PLoS One. 2016;

12. Seyfarth M, Sibbing D, Bauer I, Fröhlich G, Bott-Flügel L, Byrne R, et al. A Randomized Clinical Trial to Evaluate the Safety and Efficacy of a Percutaneous Left Ventricular Assist Device Versus Intra-Aortic Balloon Pumping for Treatment of Cardiogenic Shock Caused by Myocardial Infarction. Journal of the American College of Cardiology. 2008.

13. Karami M, den Uil CA, Ouweneel DM, Scholte NT, Engström AE, Akin S, et al. Mechanical circulatory support in cardiogenic shock from acute myocardial infarction: Impella CP/5.0 versus ECMO. Eur Hear J Acute Cardiovasc Care. 2020;

14. Kovack PJ, Rasak MA, Bates ER, Ohman EM, Stomel RJ. Thrombolysis plus aortic counterpulsation: Improved survival in patients who present to community hospitals with cardiogenic shock. J Am Coll Cardiol. 1997;

15. Manzo-Silberman S, Fichet J, Mathonnet A, Varenne O, Ricome S, Chaib A, et al. Percutaneous left ventricular assistance in post cardiac arrest shock: Comparison of intra aortic blood pump and IMPELLA Recover LP2.5. Resuscitation. 2013;

16. Magnus OE, Nanas J, Stomel RJ, Leesar MA, Nielsen DWT, O’Dea D, et al. Thrombolysis and counterpulsation to improve survival in myocardial infarction complicated by hypotension and suspected cardiogenic shock or heart failure: Results of the TACTICS trial. J Thromb Thrombolysis. 2005;

17. Overtchouk P, Pascal J, Lebreton G, Hulot JS, Luyt CE, Combes A, et al. Outcome after revascularisation of acute myocardial infarction with cardiogenic shock on extracorporeal life support. EuroIntervention. 2018;

18. Patel SM, Lipinski J, Al-Kindi SG, Patel T, Saric P, Li J, et al. Simultaneous venoarterial extracorporeal membrane oxygenation and percutaneous left ventricular decompression therapy with impella is associated with improved outcomes in refractory cardiogenic shock. ASAIO J. 2019;

19. Sattler S, Khaladj N, Zaruba MM, Fischer M, Hausleiter J, Mehilli J, et al. Extracorporal life support (ECLS) in acute ischaemic cardiogenic shock. International Journal of Clinical Practice. 2014.

20. Schwartz. Treating Refractory Cardiogenic Shock With the TandemHeart and Impella Devices: A Single Center Experience. Cardiol Res. 2012;

21. Schrage B, Ibrahim K, Loehn T, Werner N, Sinning J-M, Pappalardo F, et al. Impella Support for Acute Myocardial Infarction Complicated by Cardiogenic Shock. Circulation. 2019;

22. Sheu JJ, Tsai TH, Lee FY, Fang HY, Sun CK, Leu S, et al. Early extracorporeal membrane oxygenator-assisted primary percutaneous coronary intervention improved 30-day clinical outcomes in patients with ST-segment elevation myocardial infarction complicated with profound cardiogenic shock. Crit Care Med. 2010;

23. Sjauw KD, Engström AE, Vis MM, Boom W, Baan J, de Winter RJ, et al. Efficacy and timing of intra-aortic counterpulsation in patients with ST-elevation myocardial infarction complicated by cardiogenic shock. Netherlands Hear J. 2012;

24. Thiele H, Sick P, Boudriot E, Diederich KW, Hambrecht R, Niebauer J, et al. Randomized comparison of intra-aortic balloon support with a percutaneous left ventricular assist device in patients with revascularized acute myocardial infarction complicated by cardiogenic shock. Eur Heart J. 2005;

**Appendix Table S4. Characteristics of the included studies**

| **Study** | **Design** | **Inclusion/**  **exclusion criteria** | **Definition of**  **cardiogenic shock** | **Etiology of**  **shock** | **Devices**  **evaluated** | **MI**  **therapy** |
| --- | --- | --- | --- | --- | --- | --- |
| **Alushi**  **2019**  (1) | Retrospective | Inclusion: MI-CS with symptom onset ≤24 h  Exclusion: contraindication for device implantation, resuscitation lasting >30 m, LV thrombus, mechanical aortic valve, severe aortic valve stenosis, severe peripheral arterial disease precluding placement of Impella, significant RV failure, atrial or ventricular septal defect including post infarction ventricular septal defect, LV rupture and cardiac tamponade | Need for continuous administration of vasopressors for >30 m in order to maintain SBP of >90 mmHg, despite adequate fluid loading; evidence of end-organ hypoperfusion, clinical or radiological signs of PC and serum lactate >20 mg/dL | MI | Impella 2.5/4.0  (n=62)  vs.  IABP  (n=54) | PCI |
| **Anderson 1997**  (2) | Retrospective | Inclusion: MI-CS  Exclusion: previous stroke, active bleeding, previous treatment with streptokinase or anistreplase, recent trauma or major operation, non-compressible punctures | SBP < 90 mmHg for >1 h, unresponsive to fluid administration alone, thought to be secondary to cardiac dysfunction and associated with signs of hypoperfusion or a  CI <2.2 liters/min/m2 or SBP increased to 90 mmHg ≤1 h after positive inotropic drugs | MI | IABP  (n=62)  vs.  no IABP  (n=248) | Thrombo-lysis |
| **Aso**  **2016**  (3) | Retrospectivepropensity matched | Inclusion: CS treated with ECMO at admission  Exclusion: out-of-hospital cardiopulmonary resuscitation | NA | IHD  HF  Myocarditis  Valvular  Takotsubo  Endocarditis | ECMO+IABP  (n=604)  vs. ECMO  (n=1046) | NA |
| **Brunner 2019**  (4) | RCT | Inclusion: MI-CS  Exclusion: NA | SBP< 90 mmHg for >30 m or need for catecholamines to maintain a SBP >90 mmHg, clinical signs of PC, and impaired end-organ perfusion | MI | ECMO  (n=21)  vs.  no MCS (n=21) | PCI |
| **Burkhoff 2006**  (5) | RCT | Inclusion: ≥18 years-old, ≤24 h from CS onset, indwelling right catheter for measurement of PCWP and CI  Exclusion: isolated right heart failure, coagulopathy, sepsis, severe peripheral vascular disease, stroke ≤ 6 months, ≥2+ AR, ventricular septal rupture | CI <2.2 l/m2/m, MAP <70 mmHg, PCWP >15 mmHg, and  evidence of end-organ hypoperfusion or need for high-dose pressor and/or inotropic support to maintain the  patient out of CS | MI  HF | IABP  (n= 14)  vs.  Tandem Heart  (n= 19) | PCI |
| **Dziewierz 2014**  (6) | Retrospective | Inclusion: STEMI complicated by CS  Exclusion: NA | SBP < 90 mmHg, HR >100 bpm, and clinical signs of organ hypoperfusion | MI | IABP  (n=30)  vs.  no MCS  (n=21) | PCI  CABG |
| **Gu**  **2010**  (7) | Prospective | Inclusion: STEMI complicated by CS and undergoing PCI  Exclusion: mechanical complications, sepsis, significant AR, severe cerebral damage, resuscitation ≥130 m, severe peripheral vascular disease, CABG, reduced life expectancy | SBP < 80 mmHg or reduced by 120% from the baseline measurement, poor peripheral perfusion and symptoms (e.g. peripheral cyanosis, oliguria and cold extremities) | MI | IABP  (n=43)  vs.  no MCS  (n=48) | PCI |
| **Hawranek 2018**  (8) | Prospective | Inclusion: MI-CS undergoing PCI  Exclusion: unknown final TIMI flow grade status | Hypotension (SBP <90 mmHg for at ≥30 m or need for pharmacological support to maintain SBP >90 mmHg) and signs and symptoms of end-organ hypoperfusion. | MI | IABP  (n=1270)  vs.  no MCS  (n=5937) | PCI |
| **IABP-SHOCK II** (9) | RCT | Inclusion: MI-CS undergoing early revascularization (by means of PCI or CABG)  Exclusion: resuscitation >30 m, no intrinsic heart action; coma with fixed dilatation of pupils not induced by drugs; mechanical cause of CS (e.g., ventricular septal defect or papillary muscle rupture); CS onset >12 h; massive pulmonary embolism, severe peripheral arterial disease precluding insertion of an IABP, or ≥2+ AR; ≥90 years-old; etiology other than MI; life expectancy <6 months | SBP<90 mmHg for >30 m or need for catecholamines infusion to maintain a SBP >90 mmHg, clinical signs of PC, impaired end-organ perfusion | MI | IABP  (n=301)  vs.  no MCS (n=299) | PCI  CABG |
| **Impress 2017**  (10) | RCT | Inclusion: STEMI complicated by CS undergoing PCI  Exclusion: severe aorto-iliac arterial disease impeding placement of IABP or pMCS, known severe cardiac aortic valvular disease, life expectancy of <1 y, CABG ≤1 week | SBP<90 mmHg for >30 m or the need for inotropes or vasopressors to maintain a SBP >90 mmHg | MI | IABP  (n=24)  vs.  Impella 2.5  (n=24) | PCI |
| **Iqbal**  **2016**  (11) | Retrospectivepropensity matched | Inclusion: CS undergoing PCI  Exclusion: NA | SBP<90 mmHg for >30 m secondary to cardiac dysfunction, and/or the requirement for inotropic or mechanical support to maintain SBP e and adequate systemic perfusion | MI | IABP  (n=255)  vs.  no MCS  (n=475) | PCI |
| **ISAR-SHOCK**  (12) | RCT | Inclusion: MI <48 h and CS with a compromised hemodynamic state received positive inotropic drugs as needed  Exclusion: <18 years-old; resuscitation >30m; hypertrophic obstructive cardiomyopathy; definite thrombus in LV; treatment with IABP; severe valvular disease or mechanical heart valve; ventricular septal defect, acute mitral regurgitation greater than second degree, or rupture of the ventricle; predominant RV failure or the need for a RV assist device; sepsis; known cerebral disease; bleeding with a need for surgical intervention; pulmonary embolism; allergy to heparin or any known coagulopathy; ≥2+ AR; pregnancy | SBP<90 mm Hg for ≥30 m or the need for supportive measures to maintain SBP of ≥90 mmHg) and end-organ hypoperfusion (cool extremities, urine output of <30 ml/h, and HR ≥60 bpm); CI >2.2 l/m2 and a PCWP ≥15 mmHg | MI | IABP  (n=13)  vs.  Impella 2.5  (n=12) | PCI |
| **Karami 2020**  (13) | Retrospective | Inclusion: MI-CS if their systemic perfusion did not recover with inotropes, vasopressors, IABP, and/or Impella 2.5 therapy  Exclusion: revascularization with CABG, treatment with IABP or Impella 2.5, Cardiac arrest patients in whom device insertion took place during ongoing cardiopulmonary resuscitation (i.e. without return of spontaneous circulation) | SBP ≤90 mmHg for ≥30m or the need for vasopressors to maintain a SBP >90 mmHg, and other clinical signs of hypoperfusion (i.e. cold extremities, oliguria, altered mental state, elevated lactate levels, low mixed venous oxygen saturation) | MI | Impella 5.0  (n=90)  vs. ECMO  (n=38) | PCI |
| **Kovack**  **1997**  (14) | Retrospective | Inclusion: MI-CS ≤12h from symptoms onset undergoing thrombolytic therapy  Exclusion: etiology other than MI, thrombolysis started >12h after symptoms onset | SBP<90 mm Hg for >30m not responsive to fluid therapy alone, secondary to cardiac dysfunction and associated with signs of hypoperfusion  (cold, clammy skin, oliguria or altered sensorium) or a CI>2.2 liters/min/m2 | MI | IABP  (n=27)  vs.  no IABP (n=19) | Thrombo-lysis |
| **Manzo-Silberman 2012**  (15) | Retrospective | Inclusion: survivors of out of-hospital cardiac arrest with patent or predictive factors for the occurrence of post-resuscitation shock assisted by either Impella or IABP device immediately after the coronary angiogram  Exclusion: severe peripheral vascular or aortic disease; refractory out of hospital cardiogenic shock | Continuous infusion of vasopressors to maintain a SBP > 90 mmHg despite  adequate fluid loading. | Post-cardiac arrest | Impella 2.5  (n=35)  vs.  IABP  (n=43) | PCI |
| **Ohman 2005**  (16) | RCT | Inclusion: MI-CS eligible for thrombolytic therapy  Exclusion : absolute contraindication to fibrinolytic, heparin, or aspirin therapy; known internal bleeding >1month before enrollment; planned PCI; inability to insert IABP <3h after starting fibrinolysis; other known serious advanced illness likely to alter short-term prognosis; hemodynamically significant AR or stenosis; mitral regurgitation from rupture of mitral valve; ventricular septal defect; severe peripheral vascular disease; hematocrit <30%, <100,000 platelets/mm3 | Anterior MI complicated by hypotension (SBP>90 mmHg for >30 m) or any MI complicated by hypotension (SBP<100 mmHg for >30 m, heart rate >100 bpm), severe HF (Killip class III) or acute HF with hypotension (SBP<110 mmHg for > 30 m, unresponsive to fluid replacement alone, believed secondary to cardiac dysfunction, and associated with either signs of hypoperfusion-cool, clammy skin, oliguria, or altered sensorium (Killip class IV) or a CI>2.2L/min/m2 (2.5 L/min/m2 if receiving inotropic drugs) | MI | IABP + fibrinolysis (n=30)  vs.  fibrinolysis  (n=27) | Thrombo-lysis |
| **Overtchouk 2018** (17) | Prospective | Inclusion: patients presenting with refractory CS-MI treated with ECMO and undergoing coronary angiography.  Exclusion: Patients without CAD and those who did not undergo coronary angiography | Combination of LV ejection fraction <25%, CI <2.2 l/min/m2, SBP <90 mmHg for >30 m or need for catecholamines to maintain SBP >90 mmHg, and impaired organ perfusion despite a high dose of catecholamines | MI | ECMO  (n=28)  vs.  ECMO+IABP  (n=13) | PCI |
| **Patel 2018** (18) | Retrospective | Inclusion: refractory CS treated with VA-ECMO  Exclusion: NA | Combination of abnormal hemodynamics (SBP< 90mm Hg, CI <1.8 L/m/m2 without hemodynamic support or <2.2 L/m/m2 with support, PCWP >18 mmHg), metabolic derangements (pH < 7.4, lactate > 4 mmol/L), and clinical findings consistent with systemic hypoperfusion despite adequate intravascular volume resuscitation and the use of inotropic and vasoactive agents | MI | ECMO  (n=36)  vs.  ECMO + Impella 2.5/5  (n=30) | PCI  CABG |
| **Sattler 2014**  (19) | Retrospective | Inclusion: CS and successful PCI  Exclusion: technical impossibility of ECMO implantation | SBP< 90 mmHg in spite of cumulative catecholamine dosage exceeding 2.0 mg/h | MI | ECMO  (n=12)  vs.  IABP  (n=12) | PCI |
| **Schwartz 2012** (20) | Retrospective | Inclusion: CS treated with IABP or pMCS  Exclusion: NA | SBP < 90 mmHg, or if vasopressor or pMCS support was necessary to maintain SBP> 90 mmHg | MI  Cardiac arrest  HF | IABP  (n=50)  vs.  Impella 2.5  (n=7)  vs. TandemHeart (n=19) | PCI |
| **Schrage 2020** (21) | Retrospective propensity matched | Inclusion: MI-CS undergoing early revascularization (either PCI or CABG).  Exclusion: resuscitation for >30 m, no intrinsic heart action; coma with fixed dilatation of pupils that not induced by drugs; mechanical cause of CS (e.g., ventricular septal defect or papillary muscle rupture); onset of CS ≥12 h before screening; massive pulmonary embolism, severe peripheral arterial disease precluding insertion of an IABP, or ≥2+ AR, >90 years-old; etiology other than MI; life expectancy <6 months. | SBP < 90 mm Hg for > 30 m or needed infusion of catecholamines to maintain >90 mmHg, clinical signs of PC, impaired end organ perfusion | MI | IABP  (n=237)  vs.  Impella 2.5/CP  (n=237) | PCI |
| **Sheu 2010**  (22) | Prospective | Inclusion: STEMI complicated by CS.  Exclusion: left main and triple-vessel disease, acquired ventricular septal defect | Chest radiogram showing pulmonary edema with SBP <90 mmHg or persistent hypotension with SBP <90 mmHg associated with low cardiac output and clear lung fields, not related to dysrhythmia, showing no response to adequate fluid supply, and requiring vasopressor infusion.  Profound shock: SBP <75 mm Hg despite intravenous inotropic agent administration and IABP support associated with altered mental status and respiratory failure. | MI | ECMO  (n=219)  vs.  no MCS (n=115) | PCI |
| **Sjauw 2012**  (23) | Retrospective | Inclusion: STEMI patients treated with PCI complicated by CS  Exclusion: NA | SBP persistently ≤90 mmHg or vasopressors required to maintain SBP >90 mmHg, evidence of end-organ hypoperfusion (e.g. urine output <30 ml or cold/ diaphoretic extremities or altered mental status), elevated filling pressure | MI | IABP  (n=199)  vs.  no MCS  (n=93) | PCI |
| **Thiele 2005**  (24) | RCT | Inclusion: MI-CS undergoing PCI  Exclusion: >75 years-old, mechanical complication of MI, duration of CS >12h, RA failure, sepsis, significant AR, severe cerebral damage, resuscitation >30 m, severe peripheral vascular disease and other diseases with reduced life expectancy | Persistent SBP < 90 mmHg or vasopressors required to maintain SBP>90 mmHg; evidence of end-organ failure (e.g. urine output ,30 mL/h, cold skin and extremities, and serum lactate >2 mmol/L); elevated LV filling pressures (PC  or PCWP 15 mmHg); and  CI 2.1 L/m/m2 | MI | IABP  (n=20)  vs. TandemHeart (n=21) | PCI |

Abbreviations: AR: aortic regurgitation; bpm: beats per minute; CAD: coronary artery disease; CI: cardiac index; CS: cardiogenic shock; d: days; ECMO: extra-corporeal life support; h: hours; HF: heart failure; IABP: intra-aortic balloon pump; HF: heart failure; HR: heart-rate; IHD: ischemic heart disease; LV: left ventricular; m: minutes, MAP: mean arterial blood pressure, MI: myocardial infarction; MI-CS: post myocardial infarction cardiogenic shock; NA: not available; NSTEMI: non ST-elevation myocardial infarction; PC: pulmonary congestion; PCI: percutaneous coronary intervention; PCWP: pulmonary capillary wedge pressure; pMCS: percutaneous mechanical circulatory support; RV: right ventricular; RCT: randomized controlled trial, SBP: systolic blood pressure, y: years; STEMI: ST-elevation myocardial infarction.

**Appendix Table S5. Patients’ baseline characteristics**

| **Study** | **Type of device** | **Age** | **Male** | **BMI**  **(kg/m2)** | **Hypertension** | **Diabetes** | **Dyslipi-demia** | **CKD** | **Prior MI** | **Creatinine (mg/dl)** |
| --- | --- | --- | --- | --- | --- | --- | --- | --- | --- | --- |
| **Alushi 2019** | IABP | 71 (64-75) | 76 | 26 (24-29) | 63 | 37 | 70 | 15 | 13 | 1.37 (1.08-1.89) |
|  | IMPELLA | 73 (62-79) | 71 | 26 (25-29) | 58 | 51 | 58 | 19 | 18 | 1.39 (1.14-1.75) |
| **Anderson 1997** | IABP | 64 (55-70) | 68 | NA | 37 | 23 | NA | NA | 30 | NA |
|  | NO IABP | 68 (59-75) | 62 | NA | 40 | 23 | NA | NA | 27 | NA |
| **Aso**  **2016** | ECMO | NA | 69.4 | NA | NA | NA | NA | NA | NA | NA |
|  | IABP+ECMO | NA | 72.2 | NA | NA | NA | NA | NA | NA | NA |
| **Brunner 2019** | ECMO | 62 (50-68) | NA | NA | NA | NA | NA | NA | NA | NA |
|  | NO ECMO | 70 (60-74) | NA | NA | NA | NA | NA | NA | NA | NA |
| **Burkhoff 2006** | TANDEMHEART | 65.7±13.8 | 78 | NA | NA | NA | NA | NA | NA | 1.8±0.8 |
|  | IABP | 60.3±10.7 | 74 | NA | NA | NA | NA | NA | NA | 1.6±0.8 |
| **Dziewierz 2014** | NO IABP | 72 (62-81) | 38 | 26.9 (23.2-28.5) | NA | 5 | NA | 5 | 19 | NA |
|  | IABP | 64.5 (52-74) | 84 | 27.4 (24.7-29.3) | NA | 27 | NA | 0 | 27 | NA |
| **Gu**  **2010** | IABP | 70.4±7.8 | 63 | NA | 70 | 40 | 33 | 16 | NA | NA |
|  | NO IABP | 67.9±8.5 | 65 | NA | 67 | 35 | 38 | 17 | NA | NA |
| **Hawranek 2018** | NO IABP | 71.1±12 | NA | NA | 51 | 29 | 25 | NA | 22.1 | NA |
|  | IABP | 68.1±11.4 | NA | NA | 55 | 40 | 30 | NA | 22.7 | NA |
| **IABP-SHOCK II** | IABP | 70 (58-78) | 67 | 27.5 (24.7-30.1) | 72 | 35 | 41 | NA | 24 | 1.3 |
|  | NO IABP | 69 (58-76) | 71 | 26.9 (24.7-29.4) | 67 | 30 | 35 | NA | 20 | 1.26 |
| **Impress 2017** | IMPELLA | 58±9 | 75 | 25 (23-26) | 20 | 9 | 20 | NA | 5 | 96±29 |
|  | IABP | 59±11 | 83 | 26 (25-27) | 29 | 13 | 24 | NA | 4 | 102±22 |
| **Iqbal**  **2016** | NO IABP | 65 (56.75) | NA | NA | 49 | 23 | 36 | 9.3 | 16 | NA |
|  | IABP | 66 (56.75) | NA | NA | 49 | 27 | 33 | 18 | 20 | NA |
| **ISAR-SHOCK** | IMPELLA | 65 (57-71) | 62 | NA | 54 | 39 | 62 | NA | NA | NA |
|  | IABP | 67 (55-80) | 85 | NA | 69 | 23 | 54 | NA | NA | NA |
| **Karami 2020** | IMPELLA | 60±10 | 73 | 26 (24-28) | 37 | 17 | 14 | NA | 14 | 109 (90-138) |
|  | ECMO | 55±9 | 79 | 26 (25-28) | 47 | 11 | 21 | NA | 18 | 118 (88-158) |
| **Kovack 1997** | IABP | 62±12 | 59 | NA | 37 | 26 | NA | NA | 22 | NA |
|  | NO IABP | 64±13 | 63 | NA | 58 | 26 | NA | NA | 5 | NA |
| **Manzo-Silberman 2012** | IMPELLA | 57 (51-70) | 83 | NA | NA | NA | NA | NA | NA | NA |
|  | IABP | 62 (50-66) | 81 | NA | NA | NA | NA | NA | NA | NA |
| **Ohman 2005** | NO IABP | 67 (56-71) | NA | NA | NA | 11 | NA | NA | 19 | NA |
|  | IABP | 68 (57-74) | NA | NA | NA | 30 | NA | NA | 40 | NA |
| **Overtchouk 2018** | ECMO/ECMO + IABP | NA | NA | NA | NA | NA | NA | NA | NA | NA |
|  | IABP/ NO MCS | NA | NA | NA | NA | NA | NA | NA | NA | NA |
| **Patel 2018** | ECMO | 63 (50-71) | 70 | 33 (28-40) | NA | NA | NA | NA | NA | 1 (0.9-1.2) |
|  | ECMO + IMPELLA | 55(50-66) | 67 | 32 (25-34) | NA | NA | NA | NA | NA | 1.2 (0.9-1.8) |
| **Sattler 2014** | ECMO | 54.8±13.3 | NA | NA | NA | NA | NA | NA | NA | NA |
|  | IABP | 68.3±12.2 | NA | NA | NA | NA | NA | NA | NA | NA |
| **Schwartz 2012** | IABP | 67.4±11.4 | 58 | NA | 68 | 64 | 56 | 10 | 12 | 2.1±2.2 |
|  | IMPELLA | 69.7±10.5 | 86 | NA | 57 | 57 | 71 | 0 | 43 | 1.5±0.9 |
|  | TANDEMHEART | 68.5±14.1 | 74 | NA | 74 | 32 | 32 | 5 | 5 | 1.8±1.2 |
| **Schrage 2020** | NA | NA | NA | NA | NA | NA | NA | NA | NA | NA |
|  | NA | NA | NA | NA | NA | NA | NA | NA | NA | NA |
| **Sheu 2010** | NA | NA | NA | NA | NA | NA | NA | NA | NA | NA |
|  | NA | NA | NA | NA | NA | NA | NA | NA | NA | NA |
| **Sjauw 2012** | NO IABP | 61.5±13.8 | 66 | 26.6±3.7 | 31 | 17 | 34 | NA | 24 | NA |
|  | IABP | 64.7±12.7 | 68 | 26.7±3.3 | 35 | 18 | 21 | NA | 30 | NA |
| **Thiele 2005** | IABP | 65 (59-73) | 75 | NA | 75 | 55 | 45 | NA | 45 | NA |
|  | TANDEMHEART | 63 (57-70) | 76 | NA | 90 | 52 | 52 | NA | 62 | NA |

Continous variables are reported as mean ± SD or median (interquartile range), categorical variables as percentages.

**Appendix Table S6. Risk of bias assessment. Risk of bias of a) individual randomized clinical trials by Cochrane risk assessment tool and b1-b2) individual observational studies by ROBINS-I tool**


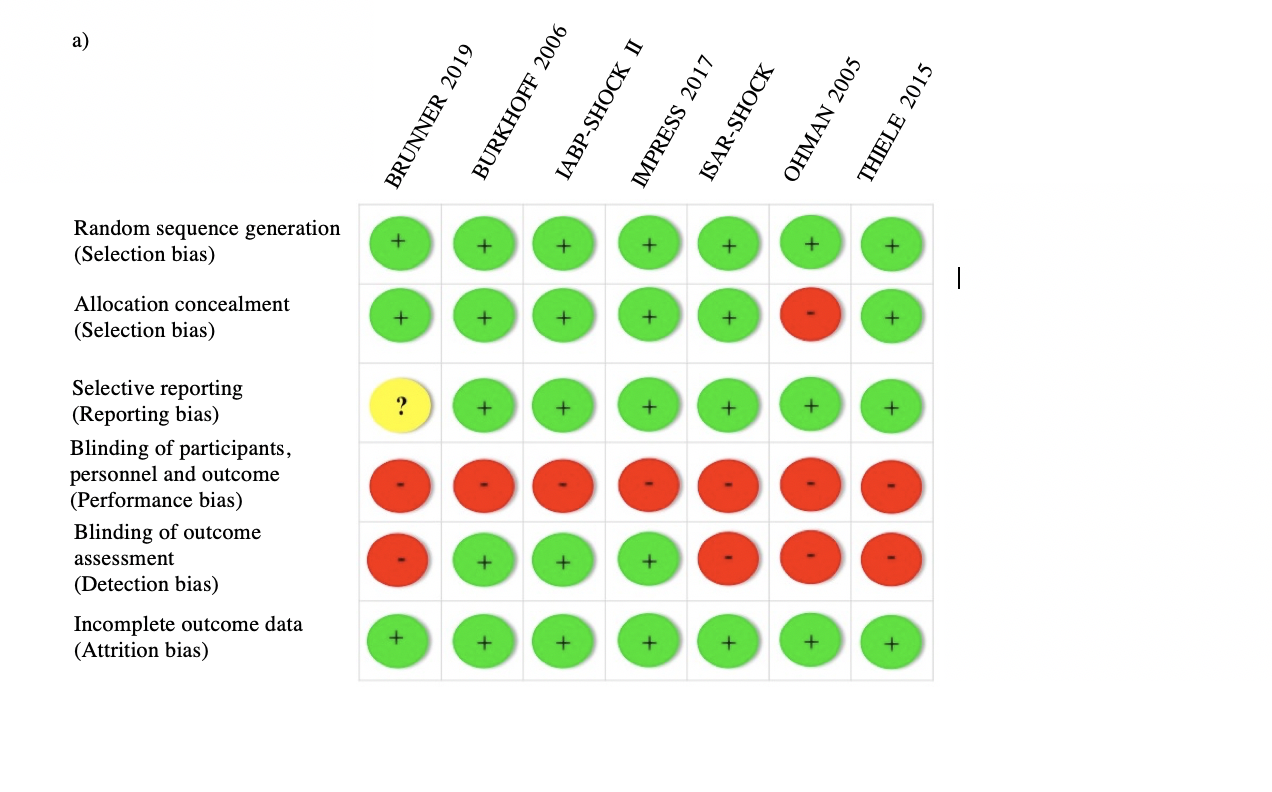


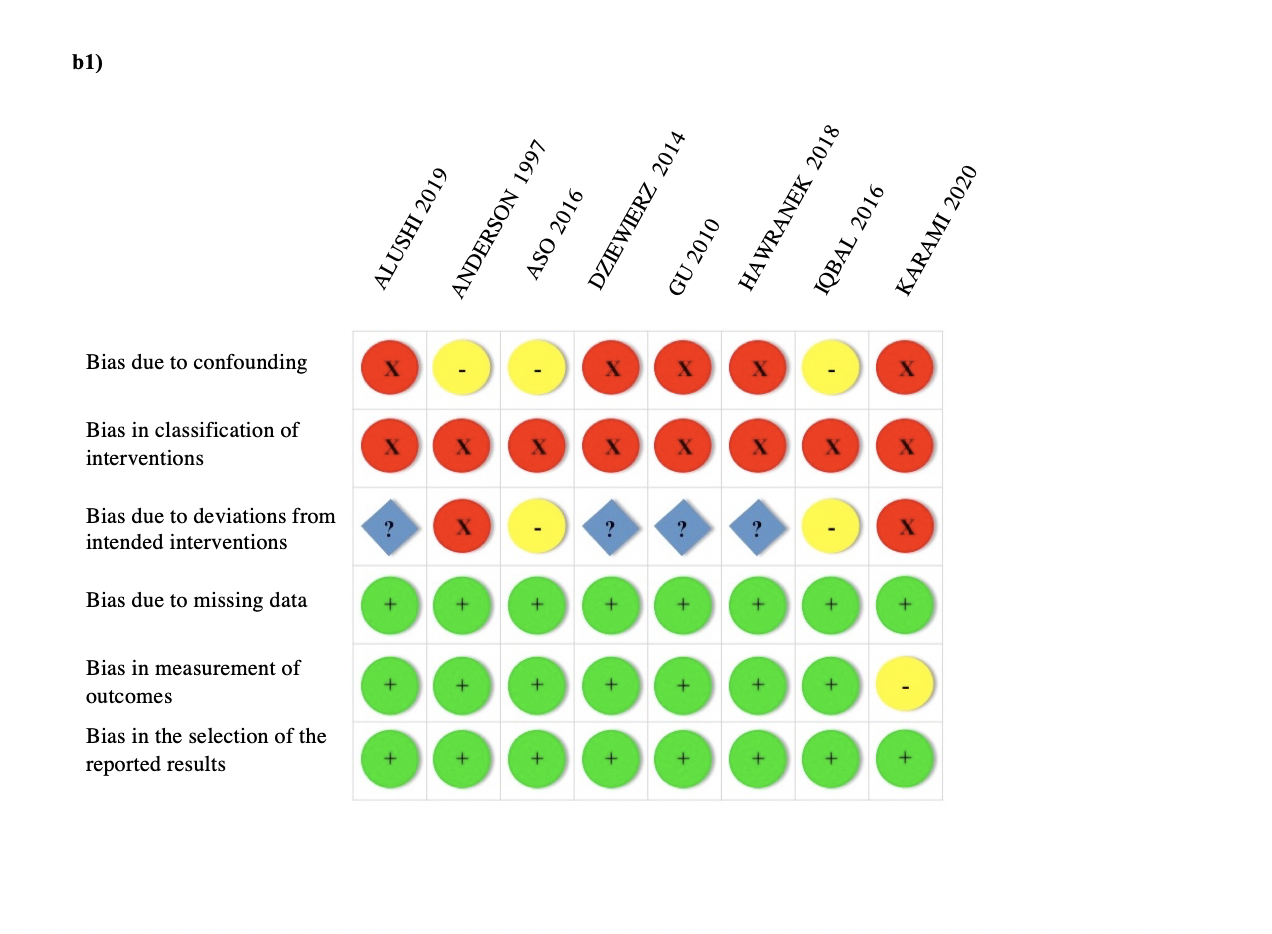


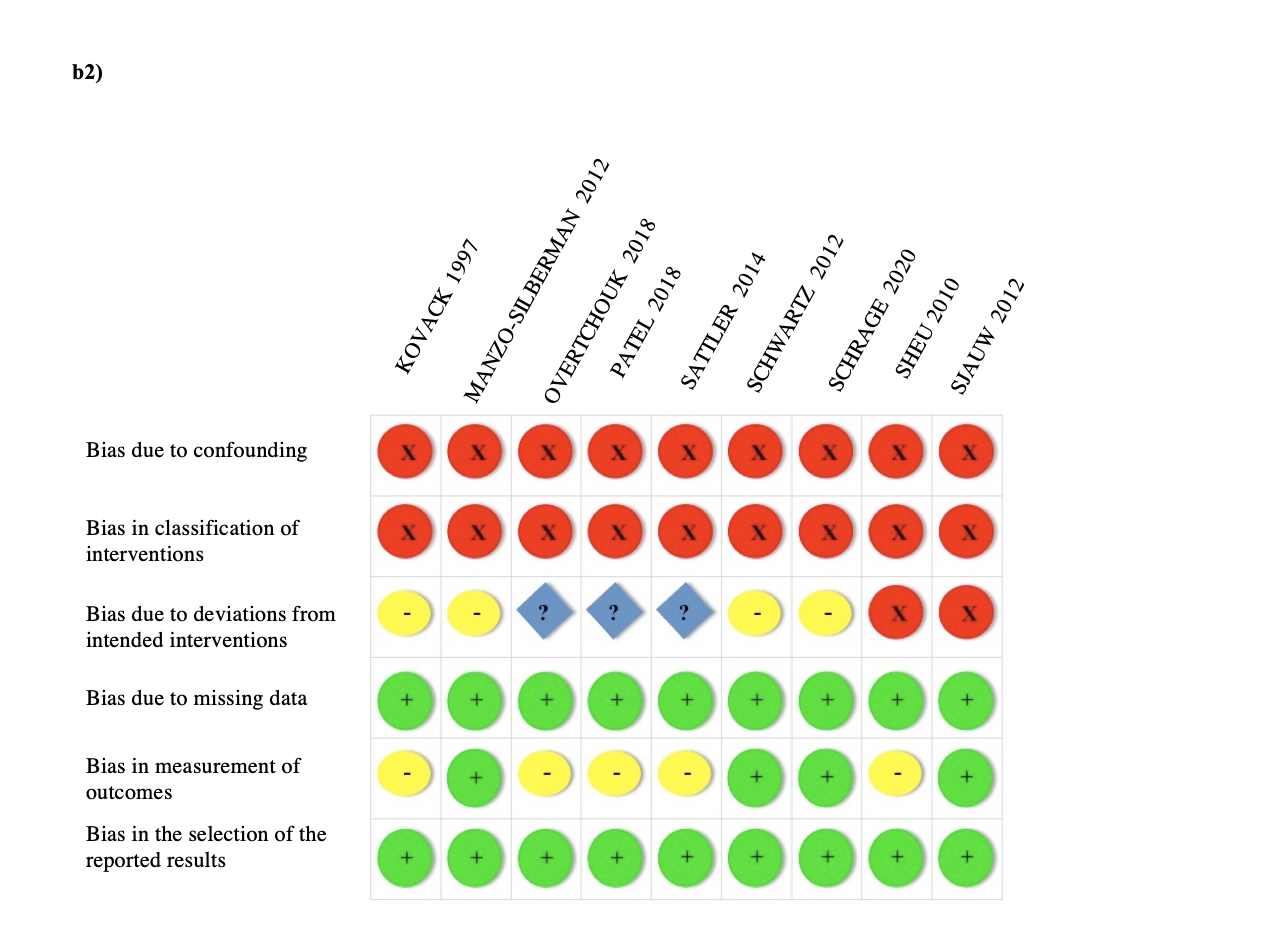


**Appendix Figure S2. Treatment networks for the secondary endpoints**

**
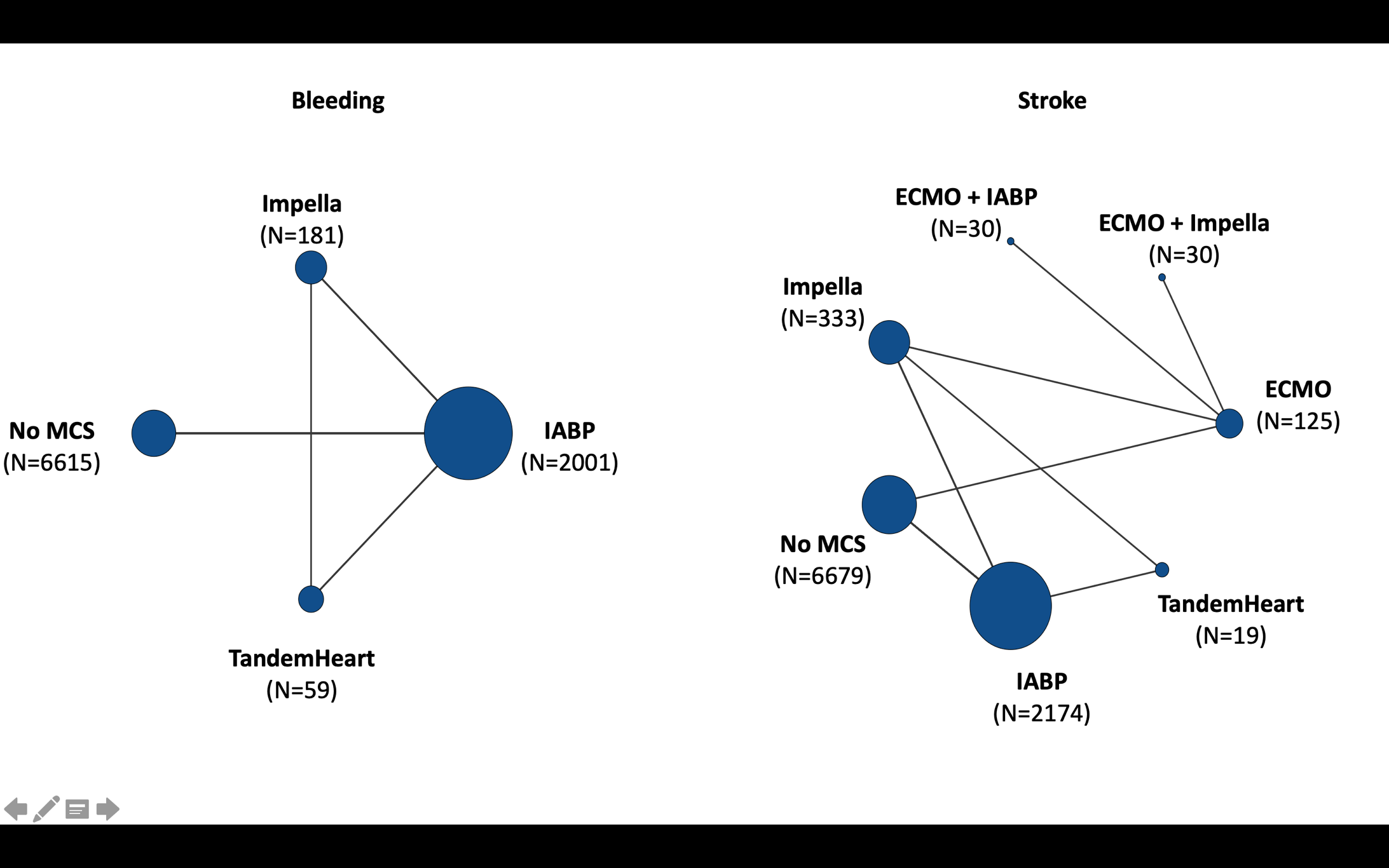
**

**Appendix Figure S3. Comparison-adjusted funnel plots and Egger’s tests**


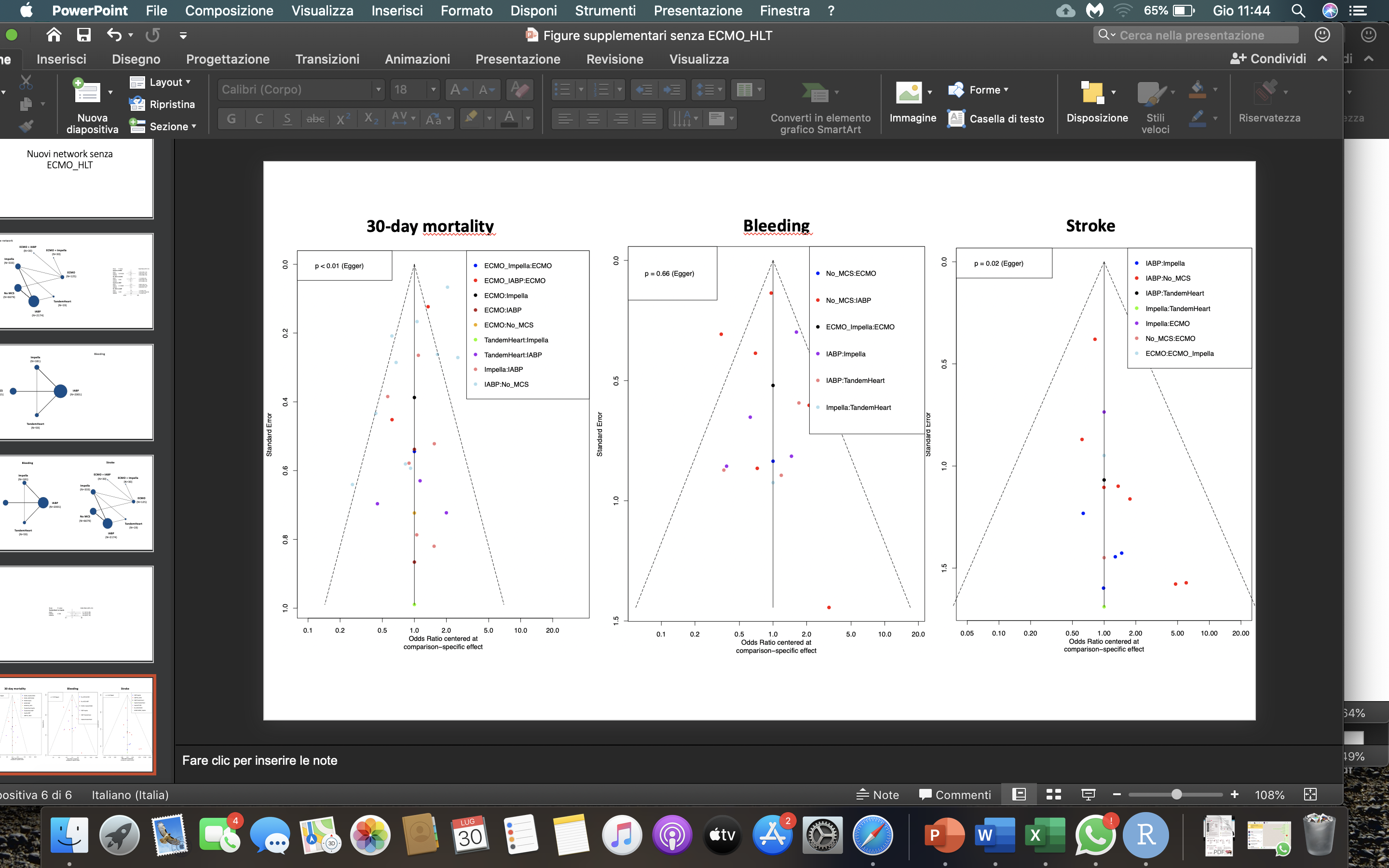


**Appendix Table S7. Per-comparisons and global heterogeneity according to I^2^**

|  | **ECMO vs. ECMO + Impella** | **ECMO vs. ECMO + IABP** | **ECMO vs.**  **no MCS** | **ECMO vs. IABP** | **ECMO vs. Impella** | **IABP**  **vs. Impella** | **IABP**  **vs.**  **no MCS** | **IABP**  **vs. TandemHeart** | **Impella**  **vs. TandemHeart** | **Global**  **I^2^** |
| --- | --- | --- | --- | --- | --- | --- | --- | --- | --- | --- |
| 30-day mortality | NA | 85 | NA | 0 | NA | 0 | 93 | 18 | NA | 88 |
| Bleeding | - | - | - | - | - | 45.9 | 80 | 13 | 0 | 69 |
| Stroke | NA | NA | NA | - | 0 | 0 | 4 | NA | NA | 0 |

**Appendix Figure S4. Node-spit analyses**


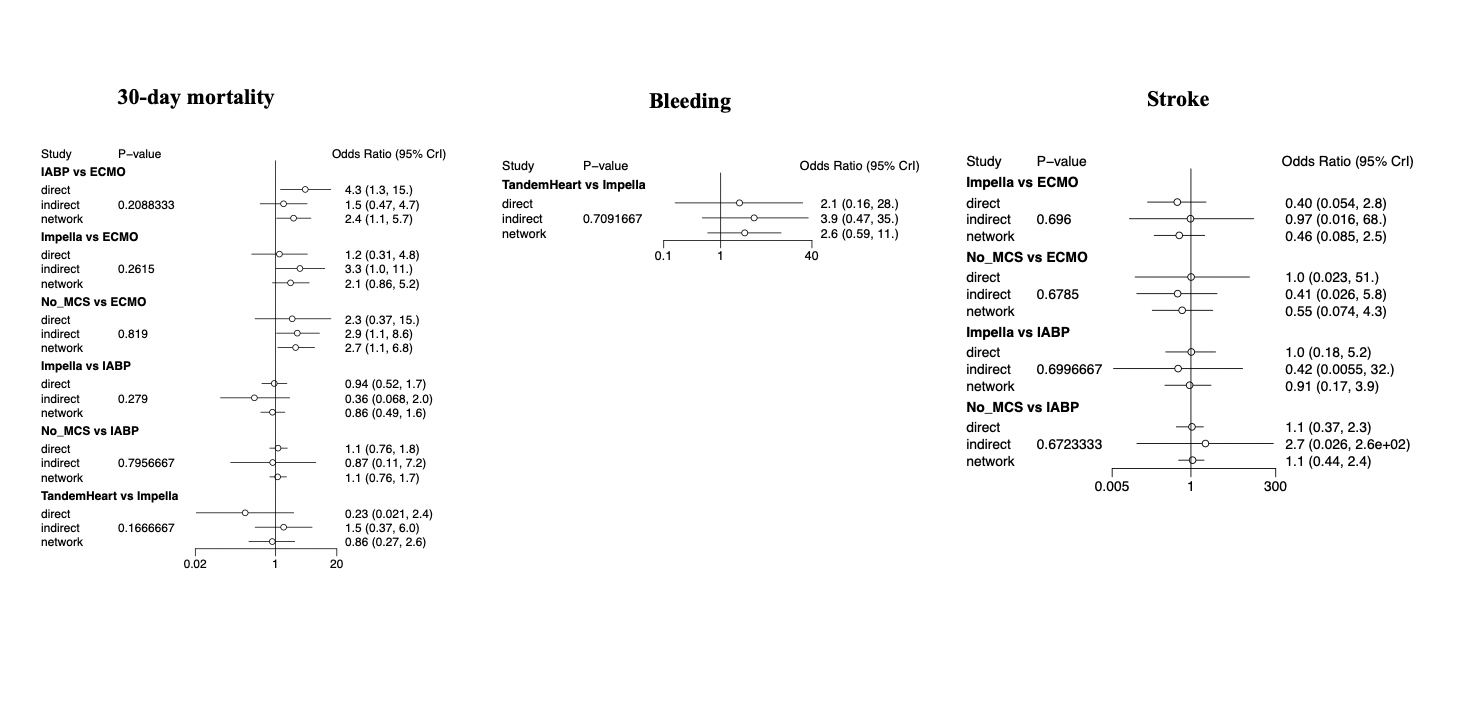


**Appendix Figure S5. Sensitivity analysis including only studies of MI-CS**

**
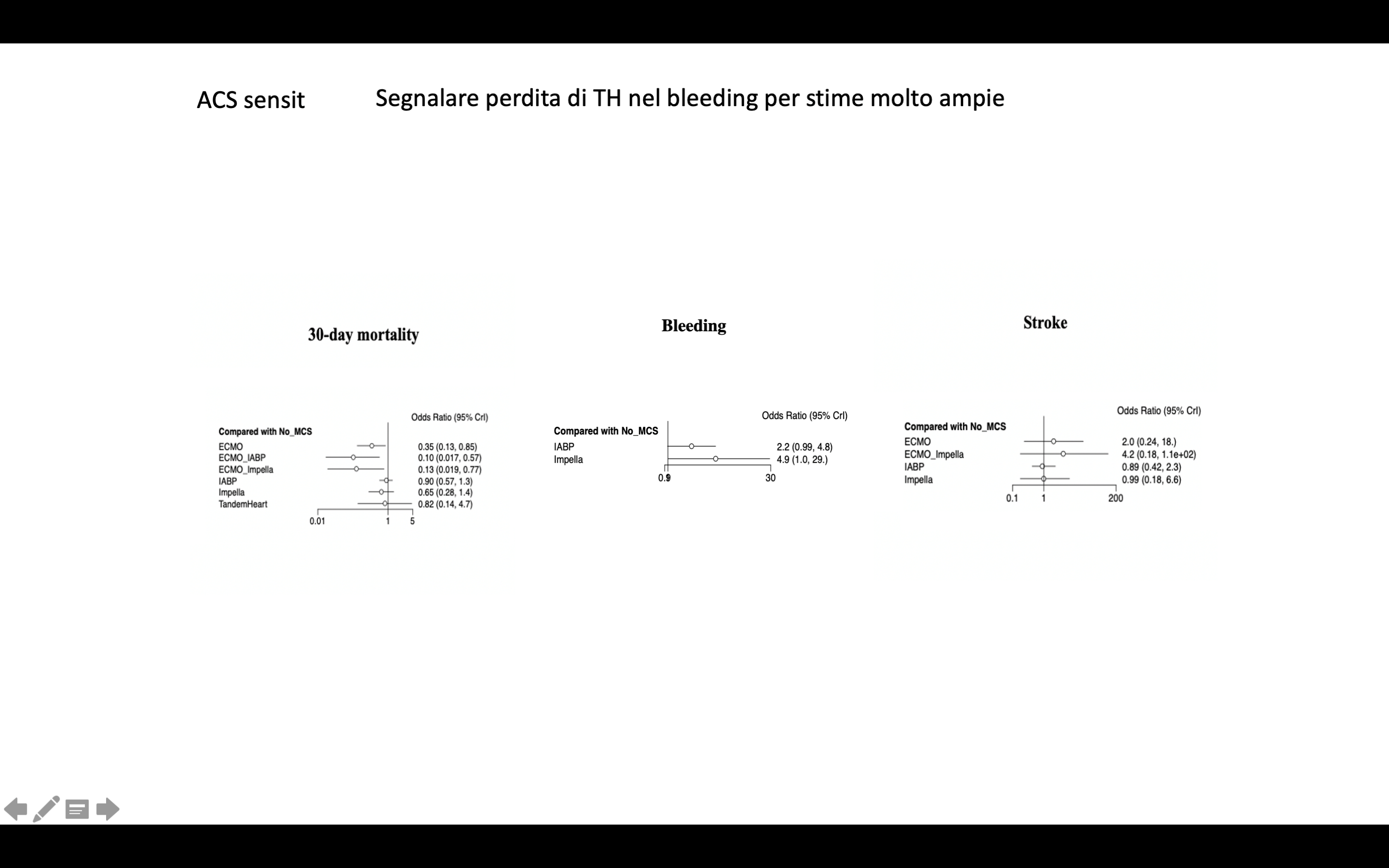
**

**Appendix Figure S6. Sensitivity analyses using the frequentist approach**


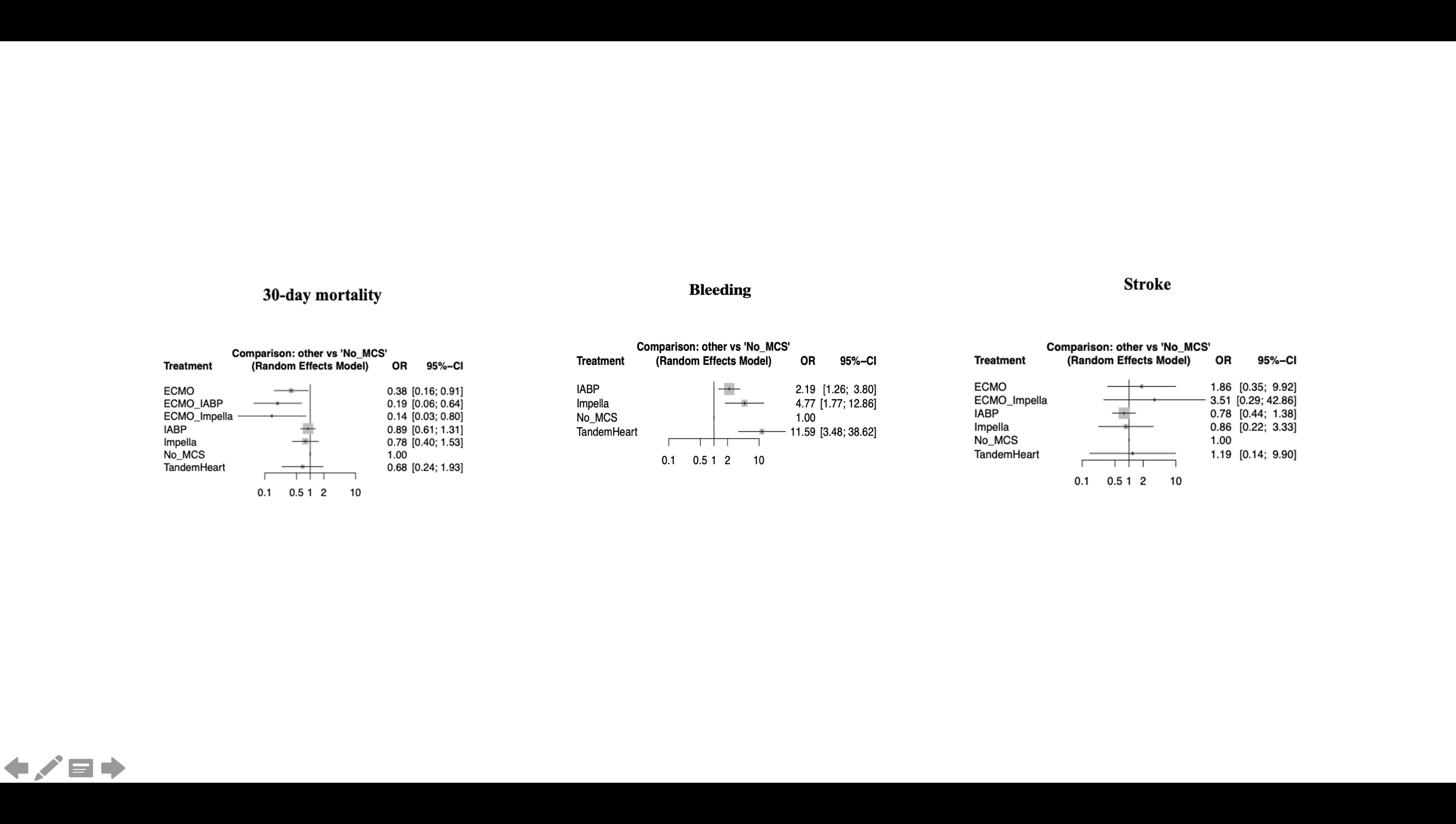


**Appendix Figure S7. Sensitivity analyses after underweighting of non-RCT studies**


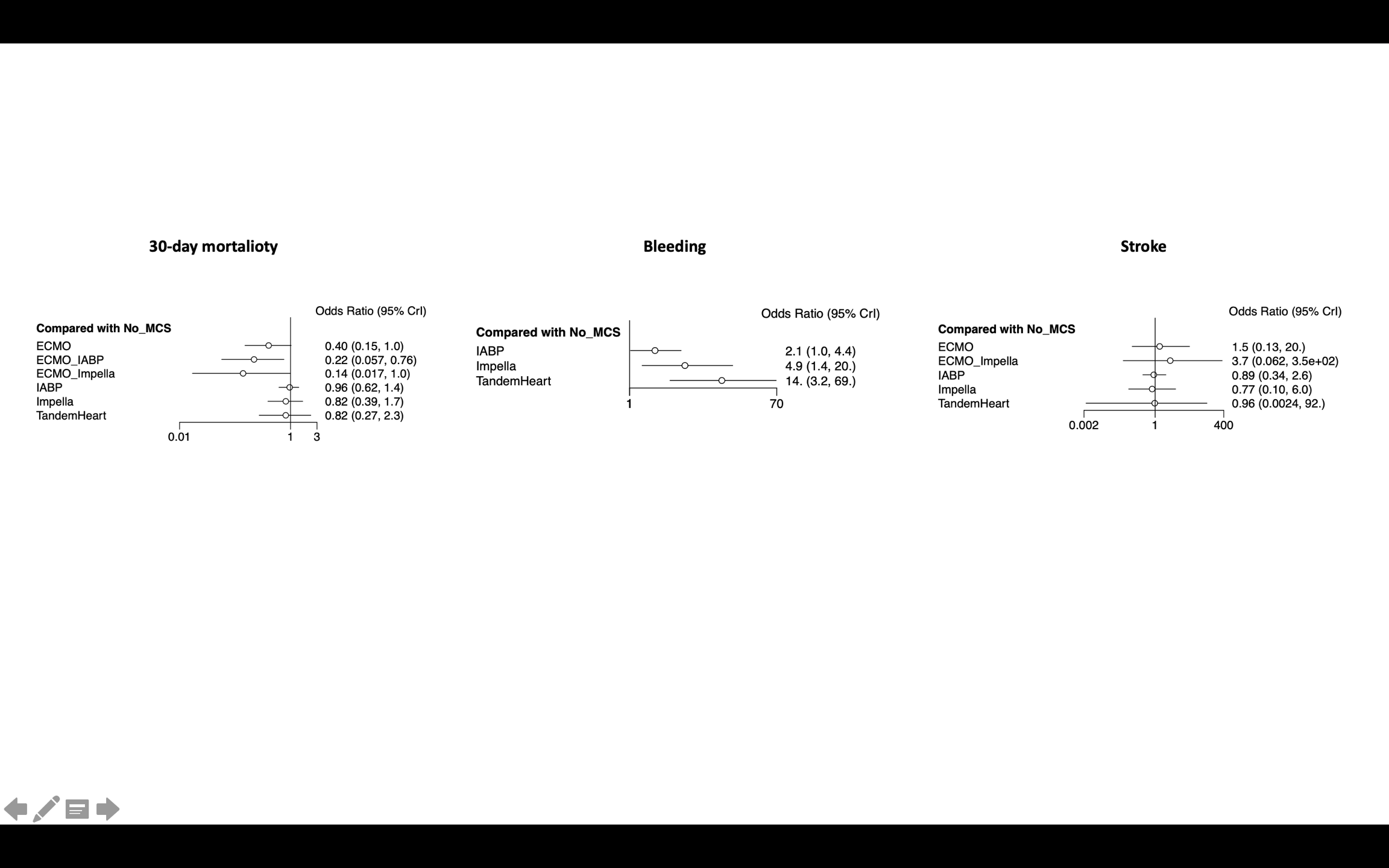

Supplement: Supplementary file 1 — Supplementary file1 (DOCX 6778 KB) [file 10741_2021_10092_MOESM1_ESM.docx]
